# Characterization of a small PlcR-regulated gene co-expressed with cereolysin O

**DOI:** 10.1186/1471-2180-7-52

**Published:** 2007-06-07

**Authors:** Julien Brillard, Didier Lereclus

**Affiliations:** 1 UMR408 Sécurité et Qualité des Produits d'Origine Végétale, INRA, Université d'Avignon, F-84000 Avignon, France; 2 Unité Génétique Microbienne et Environnement, INRA, La Minière, F-78285 Guyancourt, France

## Abstract

**Background:**

In the human pathogen *Bacillus cereus*, the expression of most extracellular virulence factors is controlled by the transcriptional activator PlcR. Among these virulence factors, cereolysin O (Clo) is an haemolysin belonging to the cholesterol-dependant cytolysins, a protein family extensively studied in Gram-positive bacteria.

**Results:**

In the genomes of bacteria belonging to the *B. cereus *group, including *Bacillus anthracis *and *Bacillus thuringiensis*, a small gene encoding a 26-amino acid peptide was present in multicopy. One copy was always found upstream from the gene encoding Clo. In *B. cereus *ATCC 14579, the small gene and the *clo *gene are co-transcribed. Transcriptional fusions showed that the three paralogues identified in this strain were expressed in a PlcR-dependent manner. We propose to name these peptides Spp for small PlcR-regulated peptides. We show that a synthetic peptide corresponding to the deduced product of the *spp *genes displayed antibacterial activity.

**Conclusion:**

The co-expression of *spp*, a small PlcR-regulated multicopy gene with *clo *suggests a yet unidentified relationship between Spp and the cholesterol-dependent cytolysin in bacteria belonging to the *B.cereus *group.

## Background

*Bacillus cereus *is an opportunistic pathogen of humans, causing local and systemic infections, and is a frequent cause of food poisoning. This species belongs to the *B. cereus *group, which includes the closely related species *Bacillus anthracis*, *Bacillus thuringiensis*, *Bacillus weihenstephanensis*,*Bacillus mycoide*s and *Bacillus pseudomycoide*s [1,2]. *B. cereus *produces several secreted proteins, including enterotoxins, cytolysins, phospholipases and proteases that may contribute to *B. cereus *pathogenicity. The expression of most of these virulence factors is controlled by the pleiotropic transcriptional activator PlcR [3,4]. This global regulator has been shown to contribute to *B. cereus *virulence in mice and insects [5] and in rabbit endophthalmitis [6]. Expression of the PlcR regulon is activated at the onset of the stationary phase of growth [7]. This activation results from cell-cell communication under the control of PapR, a small peptide that is exported, processed, and re-imported into bacterial cells in its mature form, presumably a pentapeptide, by the oligopeptide permease [8,9].

Haemolysins of the cholesterol-dependent cytolysins (CDC) family (also known as thiol-activated cytolysins) have been identified in several genera of Gram-positive bacteria [10]. These pore-forming toxins appear to play a significant role in the pathogenesis of the organisms producing them [11,12]. Listeriolysin O has been extensively studied, and this CDC has been shown to be an important virulence factor, essential for the cellulosome escape and intracellular multiplication of *Listeria monocytogenes *[13]. In *Streptococcus pyogenes*, the *spn *gene, which encodes an effector protein, is located upstream from the gene encoding Streptolysin (Slo). Cytolysin-mediated translocation involving these two proteins has been described in this bacterium [14]. In this process, Slo acts as a gate when anchored in the target-cell membrane. SPN is thus translocated into the cytoplasm of the target cell, increasing cytotoxicity [14,15]. The study of genes present in the same operons as CDC-encoding genes may therefore increase our understanding of virulence mechanisms in these bacterial pathogens.

CDC have been identified in bacteria of the *B. cereus *group. These proteins are named cereolysin O (Clo) in *B. cereus*, thuringiolysin O (Tlo) in *B.thuringiensis *and anthrolysin O (Alo) in *B. anthracis *[16-18]. We show here that three paralogous copies of an unannotated gene encoding a 26-amino acid peptide are present in the *B. cereus *ATCC 14579 genome [19]. One of these paralogues was co-transcribed with the gene encoding cereolysin O, and all three paralogues were expressed in a PlcR-dependent manner.

## Results and discussion

### Identification of a small gene, co-transcribed with *clo*

**Figure 1:**
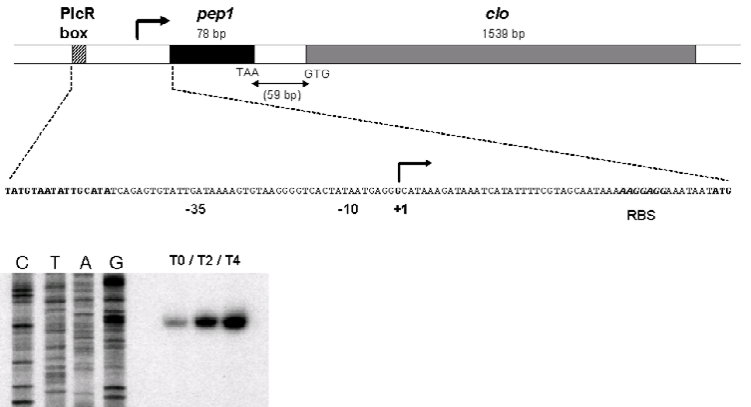
**Map of the *B. cereus *ATCC 14579 *pep-clo *locus**. (A) The PlcR recognition site (bold underlined), -10 and -35 boxes (underlined) and the putative RBS binding site (bold italic) are indicated. The transcription initiation site (+1) is indicated by the arrow. (B) Total RNA (20 μg) extracted from *B. cereus *at the onset of stationary phase (T0), two hours (T2) and four hours (T4) after T0 was subjected to primer extension analysis, using an oligonucleotide binding 65 nucleotides downstream from the Clo start codon. The same oligonucleotide was used to prime dideoxy sequencing reactions from the corresponding region obtained by PCR amplification (lanes C, T, A, G).

Small peptides often remain unannotated at the time of bacterial sequencing projects [20,21]. However, many such peptides have been shown to play a major role in bacterial physiology. Analysis of the *clo *chromosomal region of *B. cereus *ATCC 14579 revealed the presence of a 78 bp ORF between a putative PlcR box and the *clo *gene (Fig. 1a). This ORF, starting with an ATG codon, was predicted to encode a 26-amino acid peptide and was called *pep1*. It was preceded by a typical ribosome binding site at an appropriate distance.

Primer extension was carried out in order to map the transcription start site of the *clo *gene, using *B. cereus *total RNA extracted after various culture times. The 5'-end of the mRNA corresponding to *clo *was located downstream from the PlcR box, and upstream from the *pep1 *gene, indicating that a bicistronic transcript consisting of *pep1-clo *had been produced (Fig. 1b). This result suggests that *pep1 *and *clo *were co-transcribed from a single transcription start point in the conditions tested. The -10 and -35 regions of this promoter are highly similar to the -35 region (TTGACA) and -10 region (TATAAT) of vegetative promoters recognised by the σ^A ^RNA polymerase of *B. subtilis *(Fig. 1a). Similar experiments were performed with RNA extracted from the *B. cereus *Δ *plcR *strain. No signal was detected at T0, T2 and T4 in such conditions (data not shown), indicating that expression of the *pep1-clo *operon was PlcR-dependent. This result is consistent with the lack of detection of the Clo protein in the extracellular fraction of the *B. cereus *Δ*plcR *strain [4].

### Identification of *pep *paralogues and orthologues in the *B. cereus *group

**Figure 2:**
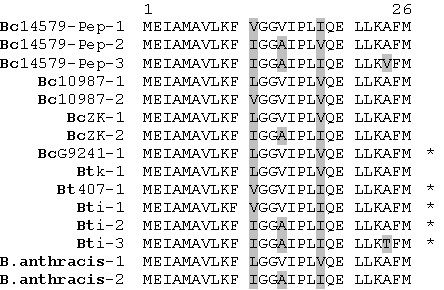
**Alignment of Pep sequences identified in members of the *B. cereus *group**. Diverging amino acids are shown in grey boxes. Alignments were performed with the Multalin version 5.4.1 program [40]. The numbers indicate copy number (1, 2 or 3) in the available genome sequences from the *B. cereus *group. Each number 1 corresponds to a Pep orthologue encoded by an ORF positioned upstream from a cholesterol-dependent cytolysin-encoding gene. Bc14579: *B. cereus *ATCC14579; Bc10987: *B. cereus *ATCC10987; BcZK: *B. cereus *EL33; BcG9241: *B. cereus *G9241; Btk: *B. thuringiensis *serovar *konkukian *strain 97-27; Bt407: *B.thuringiensis *strain 407Cry-; Bti: *B. thuringiensis *serovar *israelensis *ATCC 35646. For *B. anthracis*, the finished and unfinished genome sequences of the various strains gave the same Pep sequences, which are indicated only once. The *B. anthracis *strains tested were: strain Ames Ancestor, strain Ames, strain Sterne, strain Kruger B, strain A1055, strain CNEVA-9066, strain Western North America USA6153, strain Vollum, and strain Australia 94. In the *B. anthracis *strain A2012 unfinished genome sequence, only orthologue number 1 was identified. *: Pep orthologue identified in an unfinished genome sequence.

The deduced amino-acid sequence of the peptide encoded by *pep1 *(Pep1) was used to screen the complete genome of *B. cereus *ATCC 14579 by TBLASTN. This search led to the identification of another two paralogues elsewhere on the chromosome, not located close to any particular gene. These paralogues were called *pep2 *and *pep3*. The NCBI NR database was also screened by TBLASTN. This analysis showed that ORFs presenting strong sequence similarity with *pep1 *were identified in all the members of the *B. cereus *group (Fig. 2). In most of the completed genomic sequences, *pep1 *orthologues were found in multiple copies, up to three copies, depending on the strain. In all of the genomes in which *pep1 *orthologues were identified, one copy was located upstream from a CDC-encoding gene (*clo*, *alo *or *tlo*). Recently, the 5'-end of the *alo *transcript was mapped [22]. Despite a slightly diverging sequence between *alo *and *clo *promoter regions, the 5'-end of the *alo *transcript was positioned downstream from the PlcR box and upstream from the *pep1 *orthologue, revealing that in *B. anthracis*, a *pep1-alo *bicistronic transcript was detected, as in *B. cereus *(*pep1-clo*). Thus, the structural organisation of the operon constituted of *pep1 *and a CDC-encoding gene seems to be conserved between species of the *B. cereus *group.

No sequence displaying significant similarity to Pep1 was identified in bacteria outside the *B. cereus *group, or in other sequences in the databases, indicating that Pep1 orthologues are probably restricted to the *B. cereus *group. However, in the genome of the atypical *B. cereus *strain NVH 391–98, no *pep1 *orthologue could be identified. In this strain, the genome has a reduced size (4 Mb) compared to the other *B. cereus *group members [23], and no CDC encoding gene is present. This finding is consistent with the fact that this strain is genetically distant from other *B. cereus *group members [24].

### PlcR-dependent expression of *pep1*, *pep2 and pep3*

**Figure 3:**
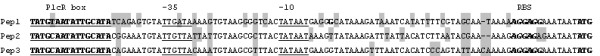
**Alignment of *spp1*, *spp2 *and *spp3 *promoter regions identified in the *B.cereus *ATCC 14579 genome**. Diverging nucleotides are shown in grey boxes. The PlcR recognition site (bold underlined), -10 and -35 boxes (underlined) and the putative RBS-binding site (bold italic) are indicated. The transcription initiation site of *spp1 *is shown in bold.

*In silico *analysis revealed the presence of a PlcR recognition site (TATGNAN_4_TNCATA) about 100 nucleotides upstream from the three *pep *genes in *B. cereus *ATCC 14579. Alignment of the upstream region of the *pep1*, *pep2 *and *pep3 *genes identified in *B.cereus *ATCC 14579 showed that the three promoter regions were very similar to the -35 and -10 regions recognised by the σ^A ^RNA polymerase of *B. subtilis *(Fig.3). A PlcR recognition site was also found upstream from all the *pep *orthologues identified in the other bacteria of the *B. cereus *group (data not shown).

**Figure 4:**
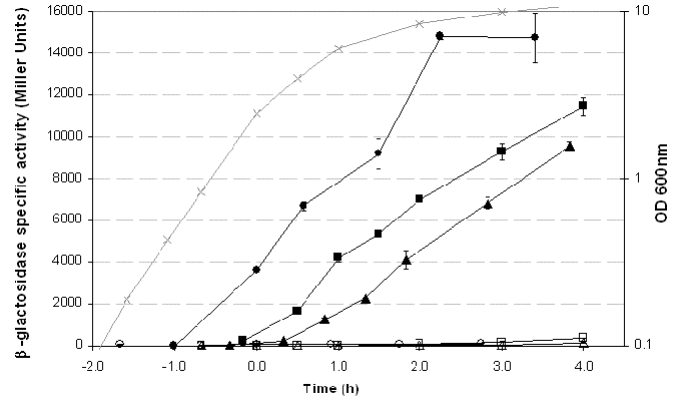
**Expression of *pep *genes in *B. cereus *ATCC 14579 and *B. cereus *ATCC 14579 Δ *plcR***. Expression of Ppep1'-Z (circle), Ppep2'-Z (square), and Ppep3'-Z (triangle) in wild-type (closed symbols) and *B. cereus *Δ *plcR *(open symbols) strains. Grey symbols, OD_600 _of cultures of bacteria. Cells were grown at 37°C in LB medium. Time zero indicates entry into stationary phase. Standard deviations of triplicate measurements are shown for β-galactosidase activity.

We investigated whether the expression of the *pep *genes in *B. cereus *ATCC 14579 depended on PlcR, by inserting about 450 bp, including each of the 5'-*pep *regions, upstream from the *lacZ *reporter gene of pHT304-18Z (Table 1). *B. cereus *strains carrying the three different recombinant plasmids were cultured in LB medium and β-galactosidase activity was measured at various stages, from the exponential growth phase to the late stationary phase (Fig. 4). The kinetics of β-galactosidase production were similar for all three strains, with *pep*-directed *lacZ *transcription activated at the end of exponential growth. However, transcription from the *pep1 *promoter appeared to begin earlier, whereas that from the *pep3 *promoter was activated later. These slight variations in the time course of expression may reflect differences in promoter efficiency, which might result from differences in the affinity between PlcR and its target sequences. Our results indicate that all three copies of *pep *are expressed in *B. cereus *ATCC 14579. The transcriptional activity of the three *pep *promoters was drastically decreased in the *B. cereus *ATCC 14579 Δ *plcR *mutant (Fig. 4). Thus, the expression of the three *pep *genes is PlcR-regulated. However, weak PlcR-independent expression was detected for *pep2'-Z *(below 500 Miller Units), *pep1'-Z *and *pep3'-Z *(below 100 Miller Units) (Fig. 4). This expression was significantly higher than that observed with the negative control pHT304-18Z without promoter (values < 10 Miller Units, data not shown). In *B. anthracis*, the PlcR regulator is not functional because the *plcR *gene is truncated [3]. A weak *alo *expression was detected by RT-PCR in *B. anthracis *cells grown in LB medium [25]. *alo *expression was also detected in *B. anthracis *cultured in rich media or grown in infected mice [18,22]. Thus, the weak expression of *pep1-clo *detected in *B. cereus *ATCC 14579 Δ *plcR*, may be similar to the *alo *expression observed in *B. anthracis*, which does not produce an active PlcR molecule. These peptides were designated Spp, for small PlcR-regulated peptide.

**Table 1:** Strains and plasmids used in this study

Strain or plasmid	Relevant genotype	Source or reference
Strains		
*B. cereus *ATCC14579		laboratory collection
*B. cereus Δ plcR*	ATCC14579 *plcR::Km*	[5]
*B. subtilis *168		laboratory collection
*B. cereus *F4430/73		laboratory collection
*B. thuringiensis *407 Cry-		laboratory collection
*E. coli *ET12567	(*F*^- ^*dam-13*::Tn*9 dcm-6 hsdM hsdR recF143 zjj-202*::Tn*10 galK2 galT22 ara14 pacY1 xyl-5 leuB6 thi-1*)	laboratory collection
*Proteus mirabilis*		N. Boemare
*Pseudomonas aeruginosa*		N. Boemare
*Salmonella spp*.		N. Boemare
*Enterococcus faecalis*		P. Serror
*Listeria innocua*		laboratory collection
*Streptococcus agalactiae*		P. Serror
*Staphylococcus aureus*		P. Serror
		
Plasmids		
pHT304-18'Z	Ap^R ^and Em^R ^cloning vehicle; *lacZ *reporter gene	[38]
pHT-Ppep1'Z	433 bp region upstream from *clo *start codon inserted between *Pst*I and *Bam*HI sites of pHT304-18'Z	this work
pHT-Ppep2'Z	448 bp region upstream from *pep2 *start codon inserted between *Hin*dIII and *Bam*HI sites of pHT304-18'Z	this work
pHT-Ppep3'Z	480 bp region upstream from *pep3 *start codon inserted between *Hin*dIII and *Bam*HI sites of pHT304-18'Z	this work

Km, kanamycin; Ap, ampicillin; Em, erythromycin.

### Putative role of the Spp peptides

*Bacillus *species are known to produce and export an abundance of small peptides. Several of these peptides are involved in signalling or have antimicrobial activity [21]. Analysis of the deduced amino-acid sequence (26 aa) of *spp1 *(*pep1*) with the SignalP 3.0 server showed there to be no predicted signal peptide. However, a double-glycine motif was found at positions 12 to 13 in all the Spp orthologues (Fig. 2). This double-glycine motif is a characteristic of some secreted peptides, such as competence-stimulating peptides in streptococci and bacteriocins in lactic acid bacteria [26]. The leader region of such peptides is cleaved after the double-glycine motif by an ABC transporter [27]. The presence of the double-glycine motif suggested that Spp is exported. By analogy to the described functions of double-glycine peptides in other Gram-positive bacteria, and given that competence has never been described in *B. cereus*, we hypothesized that Spp has a bacteriocin-like function.

**Figure 5:**
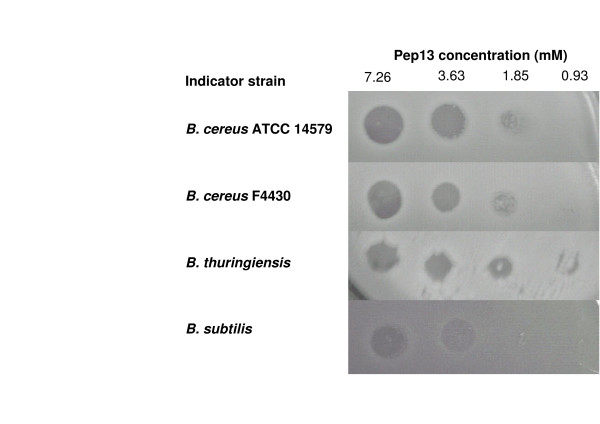
**Agar-spot tests showing antibacterial activity of synthetic Pep13**. Indicator strains were grown in LB to an OD_600_= 0.6 and diluted to OD= 0.2 before spreading on LB agar. We spotted 15 μl of Pep13, at dilutions of 7.26 mM to 0.93 mM, on indicator strains. Antibacterial activity was assessed after overnight incubation at 37°C.

For analysis of the physiological role of Spp, the entire deduced amino-acid sequence of s*pp1 *(26 aa), and the 13 aa C-terminal region of this peptide (starting after the two glycines) were synthesised chemically, giving Pep26 and Pep13, respectively. These two molecules were tested against various target bacterial cells, to determine whether Spp1 had bacteriocin-like functions. No growth inhibition was observed with the negative control (diluted DMSO) for any bacterial cell (not shown), whereas Pep13 displayed antibacterial activity at high concentrations (7.26 mM) on *Bacillus *target cells: *B. subtilis*, *B. thuringiensis, B. cereus *F4430, and *B.cereus *ATCC 14579 (Fig. 5). The antibacterial activity of Pep13 was detectable at dilutions down to 1.85 mM. Pep13 (at 7.26 mM) also displayed antibacterial activity against other Gram-positive target bacteria: *Enterococcus faecalis*, *Streptococcus agalactiae *and *Listeria innocua*, but not against *Staphylococcus aureus *(data not shown). We also assayed activity against Gram-negative indicator bacteria: Pep13 (at 7.26 mM) displayed weak antibacterial activity against *Salmonella *spp., but not against *Escherichia coli *K12, *Proteus mirabilis *or *Pseudomonas aeruginosa *(data not shown). Antibacterial activity of Pep26 (at 2.45 mM) resulted in only a small growth inhibition zone in assays with *Bacillus *indicator cells (data not shown), and no effect was observed against other indicator bacteria. The C-terminal region of Spp1 (synthetic Pep13) had stronger antibacterial activity than the entire Spp1 molecule (synthetic Pep26). This suggests that processing by cleavage downstream from the double-glycine motif may be necessary for peptide activation.

Among the indicator strains tested, *B. cereus *strains which are Spp producers, were the most affected by the Pep13 antibacterial activity. Thus, other maturation process such as posttranslational modifications, are probably required to protect the bacterial cells against their own peptide.

When *B. cereus *vegetative cells were incubated 1 hour in a phosphate buffer supplemented with Pep13 (to a final concentration of 0.7 mM), the number of CFU decreased from 1.5 (+/-0.1) × 10^7^/ml to 3.3 (+/- 0.7) × 10^6^/ml (experiments were repeated twice). In the same conditions, the number of *B. subtilis *CFU decreased from 2.1 × 10^7^/ml to 2.3 × 10^5^/ml. This indicates that Pep13 was bactericidal rather than bacteriostatic against these target cells.

However, although *spp *is expressed, there is no evidence that Spp is actually synthesized and secreted. Furthermore, given the high concentrations of Pep13 required in our assays, we cannot rule out that the antibacterial activity detected is caused by the high Pep13 hydrophobicity rather than by a specific antibacterial activity.

*B. cereus *has been isolated from soil, and from the gut of insects and nematodes [28]. Like many other bacteria isolated from such ecological niches in which there is strong competition between numerous species of micro-organisms for colonisation, *B.cereus *has been shown to produce antimicrobial peptides [29,30]. Recently, an antibacterial substance with a molecular mass of 3.4 kDa, active only against Gram-positive bacteria, was described in *B. cereus *ATCC 14579 [31]. This antibacterial activity is probably not caused by Spp, because its antibacterial spectrum is different and the predicted molecular mass of Spp1 is lower: 2.9 kDa (26 aa), and 1.5 kDa for the C-terminal fragment of Spp1 (13 aa). However, we cannot rule out the possibility that Spp1 undergoes post-translational modifications, accounting for differences in molecular mass and antibacterial spectrum.

Two small peptides with double-glycine leader sequences produced by competent cells of *S. pneumoniae *were recently shown to be involved in the lysis of non-competent *S.pneumoniae *cells, leading to the release of pneumolysin, a non-secreted CDC. This work revealed the existence of co-operation between bacteriocins and a CDC [32]. In *B. cereus*, Clo, which is found in the extracellular fraction [4], is most probably exported by the SEC machinery because it has a signal peptide. Thus, the link between Spp and Clo is probably different from that described in *S. pneumoniae*.

In *S. pyogenes*, a co-operative effect between a CDC (Slo) and a protein (Spn) encoded by a gene from the same operon has been observed. This co-operative effect increases toxicity to target cells [14]. We showed that *spp1 *(*pep1*) and *clo *are co-transcribed in *B. cereus *ATCC 14579. This operon structure was found to be conserved among bacteria belonging to the *B. cereus *group. These findings suggest that Clo and Spp might co-operate to play a role similar to that of Slo and Spn in *S.pyogenes*, in specific ecological niches or growth conditions that remain to be determined.

## Conclusion

This work has led to the identification of *spp *genes present in all members of the *B. cereus *group. We showed that the three *spp *genes of *B. cereus *ATCC14579 were expressed in a PlcR-dependent manner. In all the *B. cereus *group strains, a *spp *gene is coexpressed with the CDC genes encoding cereolysin, thuringiolysin or anthrolysin. The biological signification of this co-expression and the proposed Spp antibacterial role will have to be clarified.

## Methods

### Strains and growth conditions

The strains used in this study are listed in Table 1. *E. coli*, and *B. cereus *cells were routinely grown in Luria broth (LB), at 37°C with vigorous shaking. The antibiotic concentrations used for bacterial selection were: ampicillin, 100 μg.ml^-1^; erythromycin, 10 μg.ml^-1 ^and kanamycin, 150 μg.ml^-1^. Bacteria with the Lac^+ ^phenotype were identified on LB agar containing 40 μg.ml^-1^X-Gal.

### Database comparison and sequence analysis

TBLASTN alignments were performed with the deduced amino-acid sequence of the protein encoded by *pep1 *from *B. cereus *ATCC 14579 to screen the NR database [33]. The putative signal peptide in the polypeptide sequence was identified with the SignalP 3.0 server [34].

### DNA manipulation

Plasmid DNA was purified from *E. coli *using QIAprep spin columns (Qiagen). Chromosomal DNA was extracted from *B. cereus *cells as previously described [35]. Restriction enzymes and T4 DNA ligase were used as recommended by the manufacturer (New England Biolabs). Oligonucleotide primers were synthesised by Proligo-Genset (Paris, France). PCR was performed in a GeneAmp PCR system 2400 thermocycler (Perkin-Elmer), using the high-fidelity Pfx DNA polymerase (Invitrogen). Amplified DNA fragments were purified with the QIAquick PCR Purification Kit (Qiagen), digested and separated on 0.7% agarose gels. Digested DNA fragments were extracted from agarose gels by centrifugation in a filter device (Ultrafree DA, Millipore). All constructs were verified by DNA sequencing (GenomeExpress, France). Electroporation was used to transform *E. coli *and *B. cereus*, as previously described [36,37].

### Construction of *pep'-lacZ *transcriptional fusions

We constructed *pep'-lacZ *transcriptional fusions by inserting a PCR-amplified DNA fragment harbouring the putative *pep1, pep2 *or *pep3 *promoter regions, digested at the endonuclease sites introduced in the primers (Table 2), between the corresponding sites of pHT304-18'Z [38]. The recombinant plasmids (Table 1) were introduced into *B. cereus *ATCC 14579 wild-type and Δ *plcR *mutant strains by electroporation.

**Table 2:** Primers used

Primer name	5'-3' sequence*	Restriction sites
Ppep1-L	GATACTGCAGCCTTATGGGCCAATAGCAGT	*Pst*I
Ppep1-R	CGTCGGATCCTGATTGATAAATGATTGCTAACTAA	*Bam*HI
Ppep2-L	CGCAAGCTTTCTAAACAAGGAATCCTACAAAG	*Hin*dIII
Ppep2-R	CGCGGATCCCTCCTCTTTTTCGTATTAAGATG	*Bam*HI
Ppep3-L	CGCAAGCTTGGAAATAGTGGGTCTAGAACAT	*Hin*dIII
Ppep3-R	CGCGGATCCCCTCTTTTGTTAATACTGGGA	*Bam*HI
Extsnclo	CTAACTAATAAACATGCAAGGAAC	

* Restriction enzyme sites are underlined.

### β-Galactosidase assay

β-Galactosidase specific activities from cells of *B. cereus *strains harbouring plasmids with *lacZ *transcriptional fusions were measured as previously described [35], and were expressed in units of β-galactosidase per milligram of protein (Miller units). The Bradford method (BioRad protein assay) was used for total protein quantification.

### RNA extraction and primer extension

Total RNA was extracted from *B*. *cereus *ATCC 14579 wild-type and Δ*plcR *cells grown in LB at 37°C, at the onset of stationary phase (T0), two hours (T2) and four hours (T4) after T0, as previously described [39]. The *clo *transcription start site was identified by primer extension with the ExtsnClo oligonucleotide (Table 2), as previously described [39]. DNA sequencing was performed by the dideoxy chain termination method, with the same primer and the corresponding PCR product used as the template, with the T7 sequenase PCR product sequencing kit (USB Corporation).

### Antibacterial activity

The entire deduced amino-acid sequence of the *pep1 *ORF (26 aa: MEIAMAVLKFVGGVIPLIQELLKAFM), and the 13 aa C-terminal region of this peptide were synthesised chemically by Millegen (Toulouse, France). These molecules were called Pep26 and Pep13, respectively. Due to their strong hydrophobicity, these molecules were dissolved in DMSO, as recommended by the manufacturer. The resulting stock solution was then diluted with H_2_O to 7 mg.ml^-1 ^(2.45 mM) in 65% (v/v) DMSO/H_2_O for Pep26, and to 11 mg.ml^-1 ^(7.26 mM) in 25% (v/v) DMSO/H_2_O for Pep13. These solutions were further diluted in H_2_O and assayed on target bacterial cells. Indicator strains were grown in LB at 37°C with vigorous shaking, until an OD_600*nm *_of 0.6 was reached. They were then diluted in fresh LB to give an OD of 0.2 and 5 ml were spread on LB-agar plates. The plates were incubated for 10 min and excess liquid was then removed. Plates were allowed to dry at room temperature for 10 min under laminar air flow. Then, 15 μl of Pep26, Pep13, or DMSO (diluted to a final concentration of 65% as negative control) were applied to the plates inoculated with indicator strains. Plates were incubated overnight at 37°C before checking for a putative zone of growth inhibition.

In order to determine whether Pep13 was bactericidal or bacteriostatic, indicator strains were cultured as described above until an OD of 0.7 was reached. They were diluted 10 fold in a 0.1 M potassium phosphate buffer (pH 7) and 200 μl were incubated with 20 μl of a 7.2 mM Pep13 solution for 1 hour at 37°C. Then, the mixture was serially diluted to determine the number of CFU on LB agar medium.

## Abbreviations

CDC, cholesterol-dependent cytolysin.

## Authors' contributions

JB performed the experiments. JB and DL performed the data analysis, and wrote the manuscript. DL supervised the project. Both authors read and approved the final manuscript.
